# Could hybridization increase the establishment success of the biological control agent *Aphalara itadori* (Hemiptera: Aphalaridae) against invasive knotweeds?

**DOI:** 10.1002/ece3.10936

**Published:** 2024-02-08

**Authors:** Andrew Yoshimoto, Marianna Szűcs

**Affiliations:** ^1^ Department of Entomology Michigan State University East Lansing Michigan USA

**Keywords:** Bohemian knotweed, giant knotweed, host adaptation, host range, intraspecific hybridization, Japanese knotweed

## Abstract

Intraspecific hybridization between distinct populations could increase the fitness and adaptive potential of biological control agents that often have low genetic diversity and can be inbred due to long‐term laboratory rearing often at small population sizes. Hybridization can also alter host preference and performance when the parental insect populations are adapted to different host plants. We investigated the effects of hybridization between two populations (Northern and Southern) of the psyllid, *Aphalara itadori*, that have different fitness on three invasive knotweed species (Japanese, giant, and Bohemian). Fecundity, host choice, and developmental success of second‐generation reciprocal hybrids and the parental psyllid populations were compared on the three knotweed species in multiple‐choice tests. Hybridization did not increase fecundity. All three knotweed species were accepted for oviposition without preference by the Southern and the two hybrid psyllid populations. The northern psyllid population laid the most eggs on Bohemian knotweeds but those were maladaptive choices since almost all eggs failed to develop. The developmental success of the parental psyllid populations was highest on the knotweed species they were originally collected from, on Japanese knotweed of the Southern psyllids and giant knotweed of the Northern psyllids. Hybrids had intermediate or higher survival on given knotweed hosts compared to their parents. These results can inform release tactics of *A. itadori* in different regions especially where there appear to be climatic and/or host mismatches such as in Michigan. In southern Michigan, based on climate the Northern psyllid population should be released. However, the most common knotweed species in the region are Bohemian and Japanese knotweeds that do not support the development of the Northern psyllids. In this case, hybrids that may carry cold adaptations of the Northern psyllids but have better developmental success on the prevailing knotweed species may be considered for release to increase establishment success.

## Graphical abstract

Intraspecific hybrids between two host races of a biological control agent did not show hybrid vigor in terms of fecundity. One of the parental populations made maladaptive oviposition choices that did not support offspring development. The hybrids had intermediate developmental success on three knotweed species that could make them suitable for field release in different regions.

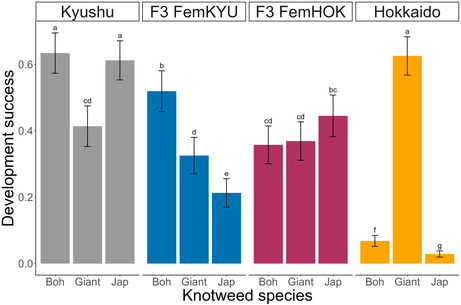

## 1 INTRODUCTION

Classical biological control aims to introduce and establish exotic natural enemies to reduce populations of invasive species. It can provide long‐term and sustainable control of invasive species; however, low establishment success and variable or low impact of the introduced agents are ongoing problems during the implementation of biocontrol programs (Cock et al., 2016; Schwarzländer et al., 2018; Van Driesche et al., 2020). This could be due to a variety of factors, including reduced genetic diversity because of long‐term laboratory rearing that can limit the ability of the biocontrol agents to adapt to their new environment (Szűcs et al., 2019). The use of intentional intraspecific hybridization as a tool, and in general, better integration of evolutionary principles into the practice of biological control are emerging new directions to enhance the fitness and adaptive ability of biocontrol agents (Leung et al., 2020; Moffat et al., 2021; Sentis et al., 2022; Szűcs et al., 2019).

Hybridization can promote rapid evolutionary change and facilitate adaptations since it can increase genetic variation, which serves as the raw material for evolution (Dlugosch et al., 2016; Schierenbeck & Ellstrand, 2009; Stebbins, 1959). Hybridization can also create novel genotypes that may exhibit unique traits and adaptations that are not found in the parental populations (Arnold, 1997; Rieseberg & Willis, 2007; Stebbins, 1959). Early‐generation hybrids can exhibit hybrid vigor, or heterosis, whereby the fitness of hybrids is higher than that of the parents (Edmands, 2007; Lynch, 1991). However, recombination in later‐generation hybrids can erase heterosis, disrupt co‐adapted gene complexes (hybrid breakdown), and outbreeding depression may ensue that can result in lower fitness of hybrids than their parents (Edmands, 2007; Lynch, 1991). The effects of hybridization can depend on the genetic distance between individuals (Edmands, 2007) and crossing between closely related populations may not result in significant differences in the traits of hybrids.

In the context of biological control, promoting rapid adaptation to novel and changing climates is desirable, but there are concerns that hybridization may lead to rapid evolutionary changes in the host range of agents. Upon hybridization, many traits may show intermediate values compared to the parents, which may be due to the high additive genetic variance that governs many life history traits (Danilevskii, 1965; Dingle et al., 1982; Hoy, 1975; Tauber et al., 1986). However, there can also be sex‐linkage and maternal effects in the expression of traits in hybrids, which can result in resemblance toward either parent, but more often the maternal parent (Hard et al., 1993; Mousseau & Dingle, 1991; Tauber et al., 1986). From the few studies that investigated the effects of hybridization on host use, it appears that hybridization can have immediate effects on the host‐specificity of herbivorous insects. When the strains or species that are crossed have different host preferences the hybrids may exhibit specificity towards either of the parental host species and preference can change as hybridization progresses in later generations (Bitume et al., 2017; Hoffmann et al., 2002; Mathenge et al., 2010). However, when the parental strains that are crossed have similar host ranges hybridization does not necessarily alter the preference or performance of hybrids on suboptimal non‐target species (Szűcs et al., 2021). Little is known of how intraspecific hybridization may influence host and climate adaptations in herbivorous insects used as biocontrol agents and without a better understanding of these basic processes, we cannot integrate evolutionary principles into biocontrol to improve the outcomes of programs.

The current study explores whether intraspecific hybridization could be used as a tool to improve the establishment and impact of biological control agents against invasive knotweeds. Three species of invasive knotweeds, Japanese knotweed (*Fallopia japonica*), giant knotweed (*F. sachalinensis*) and their hybrid Bohemian knotweeds (*F. x bohemica*) have been targeted for biological control by the psyllid *Aphalara itadori* (Hemiptera: Aphalaridae) for over a decade in the United Kingdom, since 2014 in Canada and since 2020 in the USA and the Netherlands (Camargo et al., 2022; Grevstad et al., 2022). Despite large scale, repeated introductions of *A. itadori* using thousands of individuals locally, long‐term establishment, population growth, and control of knotweeds has not been successful at any locations to date (Fung et al., 2020; Grevstad et al., 2018, 2022; Jones et al., 2021). The lack of establishment may be due to multiple factors, including climate mismatch, predation, or low fitness of the agents because of long‐term laboratory rearing (Andersen & Elkinton, 2022; Grevstad et al., 2022; Jones et al., 2020, 2021). Given that currently, two populations of *A. itadori* are available for introduction in Michigan which are specific to different knotweed species and that the populations may have distinct climate adaptations, intraspecific hybridization between them could increase genetic diversity, improve fitness, adaptive potential to different climates, and alter host preference. These outcomes would be desirable in Michigan where only the long‐term laboratory‐reared populations are available for introduction, and where releases face the problem of matching either the best‐fitting host race on existing knotweed infestations or the best climate match of *A. itadori*.

In southern Michigan, where a humid continental climate prevails, large populations of Japanese and Bohemian knotweeds are present (misin.msu.edu). The southern population of *A. itadori* which was collected on the island of Kyushu in Japan has the best performance on these two knotweed species (Grevstad et al., 2013). However, Kyushu has a subtropical climate. The northern population of *A. itadori* was collected from a similar climate as southern Michigan, on the island of Hokkaido in Japan, but they have the best performance on giant knotweeds and low fitness on Japanese and Bohemian knotweeds (Grevstad et al., 2013). Thus, there appears to be no optimal release approach in southern Michigan using either population of *A. itadori*.

Hence, we explored the effects of hybridization on fitness, host choice, and developmental success of *A. itadori* on different knotweed species to evaluate the biocontrol potential of hybrids. We created reciprocal hybrids between the southern and northern populations and compared their fecundity and their host choices between the three knotweed species with those of the parental populations in multiple‐choice tests. We also assessed the developmental success of the hybrid and parental populations on the three knotweed species. We hypothesized that hybrids would show intermediate traits between the parental populations regarding host choice and the developmental success of the different knotweed species. In addition, we predicted that hybridization would lead to heterosis, possibly increasing the fecundity of either or both reciprocal hybrid crosses.

## 2 MATERIALS AND METHODS

### 2.1 Study system

Knotweeds were introduced in the 1800s as ornamentals and for erosion control but escaped cultivation and have now invaded 42 states in the USA, becoming particularly problematic in the northeast and Pacific Northwest (Grevstad et al., 2018). Knotweeds are herbaceous perennial plants with leathery leaves and hollow, bamboo‐like stems that can grow 1–3 m tall. They primarily reproduce vegetatively by cuttings and rhizomes (Grevstad et al., 2018). Knotweeds often create dense monocultures in forests, riverbanks, floodplains, and roadsides. They are difficult to control by chemical, physical, and cultural methods because of constant regrowth from their extensive root systems (Grevstad et al., 2018).

The knotweed psyllid, *A. itadori*, feeds on the sap of knotweeds using their piercing mouthparts. This feeding can weaken plants and at high enough psyllid densities may kill them (Grevstad et al., 2013). Psyllids undergo incomplete metamorphosis with eggs hatching into nymphs that undergo five stages that resemble adults more with each successive stage (Hodkinson, 1974). Nymphs are largely sessile, and the development time from egg to adult is about 33 days at 23°C (Shaw et al., 2009). Adult knotweed psyllids are about 2 mm long and reach sexual maturity approximately 5 days after adult eclosion but are capable of mating as early as 48 h after adult emergence. Females reach peak fecundity at 20 days old, and a single individual can lay up to 700 eggs over the course of its lifetime. Adult lifespan averages about 55 days for females and 38 days for males under ambient laboratory conditions (20°C, 50%–70% RH) (Myint et al., 2012).

### 2.2 Growing knotweeds

Rhizomes of each of the three knotweed species were collected from a single location in Michigan (Japanese knotweed: −83.495170, 42.466906, Bohemian knotweed: −83.7908, 42.6317, and giant knotweed: −88.479681, 46.6442711) in April 2021. For Bohemian knotweed rhizome was collected from a single plant so plants used in experiments would represent the same genotype. The phenotype of this Bohemian knotweed clone resembled more closely the Japanese knotweed parent in terms of leaf toughness. Rhizomes were cut into 10–15 cm pieces and transplanted into 2.5 L plastic pots using a peat and perlite potting mix (Suremix, Michigan Grower Products). Plants were fertilized with Osmocote Plus (N:P:K = 15:9:12, ICL fertilizers, USA) once every 2 months and grown at the Michigan State University (MSU) greenhouse facilities. To propagate knotweeds, 5 cm stem cuttings that contained at least one node were taken from plants of >50 cm height. Cuttings were planted similarly to the rhizomes described above and plants grown from these cuttings were used for experiments. The height of knotweed plants that were used for this experiment ranged from 20 to 35 cm.

### 2.3 Psyllid rearing and hybridization

The original Kyushu colonies were founded by individuals from collections made in 2004 and 2015 within Kumamoto prefecture on the Japanese island of Kyushu, while the source population of the Hokkaido colony originated from individuals collected in 2007 from the Lake Toya area on the island of Hokkaido (Grevstad et al., 2013). The *A. itadori* populations at MSU were founded via shipments of 480 and 400 Kyushu (southern population) adults in April and June, respectively, and 600 Hokkaido (northern population) adults in June 2021 from colonies maintained at Oregon State University. An additional 350 Kyushu individuals were shipped from the Phillip Alampi Beneficial Insect Rearing Laboratory (New Jersey Department of Agriculture) in August 2021.

We reared the Kyushu population on Japanese knotweed and the Hokkaido population on giant knotweeds (Grevstad et al., 2013) by placing 100 adults into a 40 × 40 × 60 cm mesh cage (Restcloud, Chengdu, China) containing a single potted knotweed plant for 14 days. The adults emerging over the course of 4–5 weeks were kept for an additional 2 weeks in cages to mate and then the next generation was started as described above. The rearing took place in a laboratory at room temperature (22°C ± 5, RH 40% ± 10, 16L:8D); under these conditions, psyllids developed from egg to adult in 35 days.

To create the reciprocal crosses knotweed plants infested by either Kyushu or Hokkaido 5th‐instar nymphs were cleared of all adults and monitored every 24 h. Newly‐emerging adults were collected, sorted by sex based on the identification of genitalia (Hall, 2008), and paired with similarly collected virgin individuals of the opposite sex and population. Both reciprocal hybrid crosses, the one using Kyushu females paired with Hokkaido males (FemKYU), and the one using Hokkaido females paired with Kyushu males (FemHOK) were reared on Bohemian knotweeds by placing 100 psyllids (50♀ × 50♂) in a cage containing a potted knotweed plant. Adults were kept in the cage for 2 weeks to mate and lay eggs and then removed. Developing psyllids were kept together in the same cage for 8 more weeks which allowed for adult emergence, sexual maturation, and mating. These first‐generation (F1) adults were used to start the second‐generation (F2) without any additional crossing or back‐crossing.

### 2.4 Host choice experiment

One potted knotweed plant of each species was placed into a rearing cage equidistant from each other in a randomly assigned position in each cage. Each cage was randomly assigned to receive one of four psyllid treatments (Hokkaido, Kyushu, F2 FemKYU, or F2 FemHOK), with six replicate cages set up for each treatment for a total of 24 cages. Using a manual aspirator, 25 females and 25 males about 14 days old of the corresponding psyllid population were collected in a 33 mL polystyrene vial and then released at the center bottom of the cage. The inoculation period for this experiment was 72 h, after which all adult psyllids were removed from the cage. The knotweed plants were also removed, and the number of eggs on each plant was counted by visually inspecting the surface of the leaves using a 10× magnification hand lens. Once the eggs were counted each plant was covered individually by a mesh cage (1 m tall × 40 cm wide) that was secured to the rim of the pot by an elastic band and labeled with the treatment and replication number. Individually covered plants were placed in the greenhouse (21°C ± 5, RH 50% ± 10, 16L:8D) where knotweeds were grown. After 50 days the number of emerged adults from each plant was recorded.

### 2.5 Statistical analyses

Fecundity was calculated as the sum of eggs laid within each mesh cage by the 25 females released on the three knotweed plants within each of the six replicated cages. Fecundity data were square root transformed to meet the assumptions of normal distribution. An Analysis of Variance (ANOVA) model was used with psyllid populations (Kyushu, FemKYU, FemHOK, and Hokkaido) as a fixed effect to compare the fecundity of the different populations.

To evaluate oviposition preference the number of eggs laid on each plant species by each psyllid population was compared using a linear mixed model (*lmer*) where psyllid population, knotweed species (Japanese, Bohemian, and giant) and their interactions were the fixed effects. Cage was included as a random effect to account for possible differences among plants used in the different replicates. Egg count data were square root transformed to improve normality.

A general linear mixed model (*glmer*) was used to compare *the* development success of the different psyllid populations of the three knotweed species. Given that the survival data is bounded between 0 and 1, a binomial distribution was used with a logit link function. Psyllid population, knotweed species, and their interaction were the fixed effects and the cage was the random effect in the model. Wald chi‐square test was used to obtain test statistics for this model.

For the oviposition preference and survival analyses post‐hoc, pairwise comparisons were performed using the *emmeans* package with Tukey adjustment (Lenth, 2021). All analyses were conducted in R v.4.2.3 using the *lme4* package (R Core Team, 2022).

## 3 RESULTS

**FIGURE 1:**
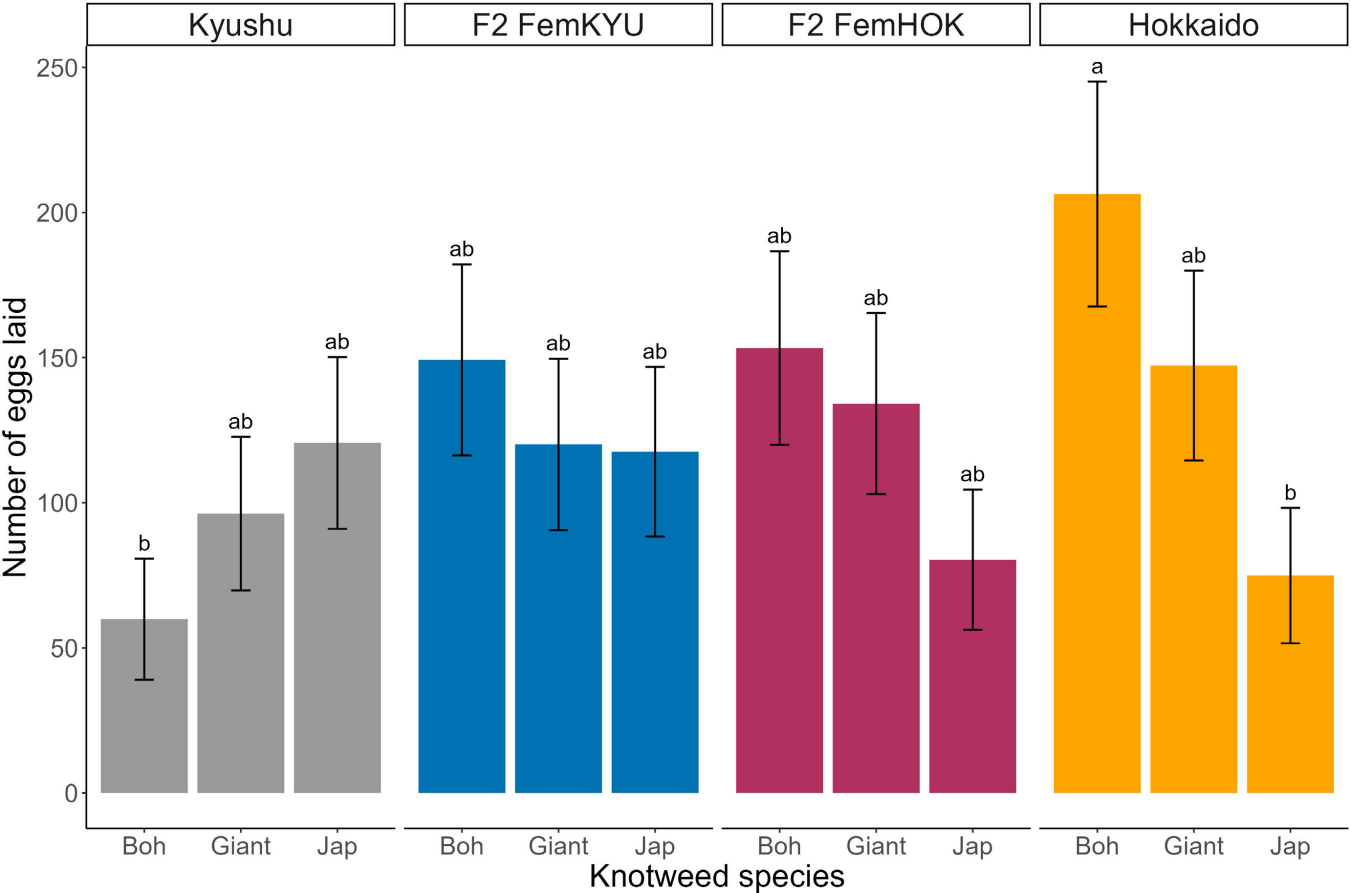
Number of eggs (mean ± SE) laid by 25 females originating from four *A. itadori* populations (Hokkaido, Kyushu, and their reciprocal hybrids: FemKYU and FemHOK) on three knotweed species (Japanese, Bohemian, and giant) over the course of 72 h in a multiple‐choice experiment. Letters above the bars indicate significant differences at (*α* < .05) across population treatments based on post‐hoc pairwise comparisons using Tukey adjustment.

There was no difference in the fecundity of the parental and hybrid *A. itadori* populations as measured for a group of 25 females (*F*_3,20_ = 1.03, *p* = .3988). The number of eggs laid by the 25 females released within each cage (replicate) summed up on the three knotweed species offered were: 316 ± 73 (non‐transformed mean ± SE) for the Kyushu parent, 456 ± 54 for the Hokkaido parent, 413 ± 83 for the FemKYU hybrid, and 378 ± 41 for the FemHOK hybrid. The different psyllid populations tended to lay a similar number of eggs (*F*_3,20_ = 1.02, *p* = .4052) on the three knotweed species (*F*_2,40_ = 2.56, *p* = .0902) except for the Hokkaido population (psyllid population × plant species: *F*_6,40_ = 2.79, *p* = .0233) (Figure 1). Hokkaido females laid significantly more eggs on Bohemian knotweeds (206.4 ± 38.7) than on Japanese knotweeds (74.9 ± 23.3) while the Kyushu parent and the hybrids did not discriminate among knotweed species.

**FIGURE 2:**
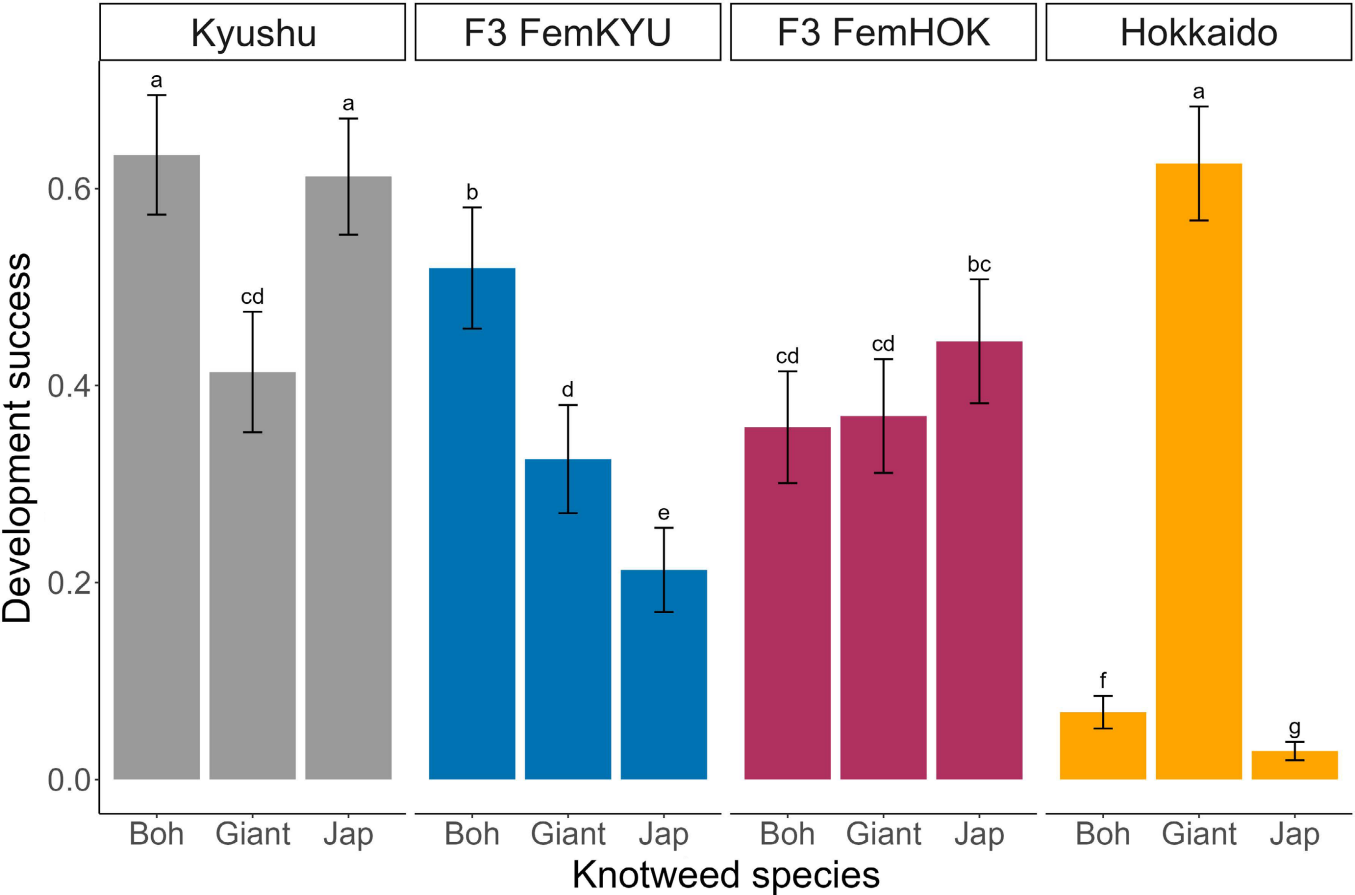
Development success (mean ± SE) of four populations (Hokkaido, Kyushu, and their reciprocal hybrids: FemKYU and FemHOK) of *A. itadori* on three knotweed species (Japanese, Bohemian, and giant) measured as the proportion of adults emerging from the number of eggs laid per plant. The letters above the bars indicate significant differences (*α* < .05) across treatments based on post‐hoc pairwise comparisons using Tukey adjustment.

There were significant differences in the development success of nymphs from the four psyllid populations on the three knotweed species (psyllid population × plant species interaction: *χ* = 833.87, df = 6, *p* < .001) (Figure 2). The FemHOK hybrid was the only psyllid population that had similar developmental success on all three knotweed species. The Hokkaido and Kyushu parent had the highest survival on their optimal hosts of giant and Japanese knotweeds, respectively, while the FemKYU hybrid performed best on Bohemian knotweed.

In the cross where the female is from the Hokkaido population and the male is from Kyushu (FemHOK) hybridization significantly increased survival on Bohemian (36%) and Japanese knotweeds (45%) compared to the Hokkaido parent (<7%). However, hybridization also led to lower development success on giant knotweeds (37%) compared to the maternal parent (63%). For the other type of cross where the female was from Kyushu (FemKYU), there was some decline in survival on Bohemian knotweeds (52%) compared to the maternal parent (63%) and a larger reduction in Japanese knotweed (21%) compared to the Kyushu parent (61%). On giant knotweed, both reciprocal crosses had similar performance as the Kyushu parent (Figure 2).

## 4 DISCUSSION

We found that the effects of intraspecific hybridization were neutral in terms of fecundity and host acceptance for a group of females. The reciprocal hybrids and the parental populations laid eggs on all knotweeds showing little preference toward any of the three species except for the Hokkaido parental population which as a group laid a greater number of eggs on Bohemian knotweed than Japanese knotweed. However, this host choice was maladaptive because of the low survival of eggs to adulthood. On the contrary, the survival of the hybrid crosses was either intermediate between the parental populations or higher than the survival of at least one of the parents on given host plants. Thus, the sum effects of hybridization appear to be either neutral or positive in this system.

We did not find evidence for hybrid vigor with regards to the fecundity of a group of females since the reciprocal hybrid populations and the parental populations laid similar numbers of eggs when summed up across knotweed species. Individual females could have exhibited hybrid vigor, but our experimental design does not allow for such evaluation. Heterosis is usually strongest in the first generation, and we used second‐generation hybrids in our experiments where the effects may be less pronounced. Yet, given that both parental populations have undergone long‐term laboratory rearing some increase in fitness was expected upon hybridization, and second‐ and later‐generation intraspecific hybrids showed higher performance than their parents in other insects as well (Hufbauer et al., 2013; Szűcs et al., 2012, 2017). It is possible that because of the small body size, high fecundity, and relative ease of rearing, *A. itadori* have been kept at large enough population sizes that buffered them from genetic problems, such as inbreeding or drift that hybridization could have alleviated. Alternatively, it is possible that the two parental psyllid populations are not genetically distinct enough to exhibit heterosis upon crossing.

When presented with a choice between three knotweed species, both the parental and hybrid *A. itadori* populations accepted all three species for oviposition (Figure 2). This indiscriminate egg‐laying behavior was also observed in a recently collected distinct population (Murakami) of *A. itadori* in paired‐choice tests (Camargo et al., 2022). Similarly, all three knotweed species were accepted for oviposition in no‐choice tests by both the Kyushu and Hokkaido populations during host range testing (Grevstad et al., 2013). All the above experiments were conducted in different geographical regions, the one using the Murakami population in the Netherlands (Camargo et al., 2022), the host range testing in Oregon and the United Kingdom (Grevstad et al., 2013), and this study in Michigan, and they all used local knotweed populations for the experiments. Thus, it appears that a variety of genotypes of the three knotweed species are all recognized as potential hosts by *A. itadori*. Thus, it should be noted that in North America most Japanese knotweed populations represent a single genotype (Gammon & Kesseli, 2010; Gaskin et al., 2014). This can be beneficial for field releases when mixed stands of different knotweed species co‐occur, however, some of these choices do not reflect the survival probability of psyllids.

The host choices of the Hokkaido population appear maladaptive since at least some of the females from this population laid most of their eggs on Bohemian knotweed but can only develop on giant knotweed, which may be an artifact of over a decade of laboratory rearing and lack of exposure to different knotweed species. The developmental success of Hokkaido psyllids on Bohemian and Japanese knotweeds was under 1% (Figure 2). The phenotype of the Bohemian knotweed clone used in our experiments resembled more closely the Japanese knotweed parent. This could partly explain the poor developmental success of Hokkaido psyllids. Such suboptimal host choice behavior has been observed in many other herbivorous insects (Alred, 2021; Badenes‐Perez et al., 2006; Berenbaum, 1981; Davis & Cipollini, 2014; Faldyn et al., 2018; Ries & Fagan, 2003; Schlaepfer et al., 2005). Notably, monarch butterflies (*Danaus plexippus*) may lay up to a quarter of their eggs on invasive swallow‐wort vines (*Vincetoxicum* spp.) that are related to their milkweed hosts but do not support larval development (Alred, 2021; Casagrande & Dacey, 2014). The Kyushu population does not discriminate among knotweed species for oviposition, but it has relatively high development success (40%–62%) on all three species. Our results for survival of both the Kyushu and Hokkaido populations on the different knotweed species are in line with findings during host‐specificity testing (Grevstad et al., 2013).

Our development success results align with one other study by Fung et al. (2020) that compared the survival of the F4 FemHOK cross and the Kyushu parent on Japanese knotweed and found lower performance of the hybrids. The conclusion made by Fung et al. was that hybridization would not be beneficial for biological control in this system. However, we can place the effects of hybridization of *A. itadori* in a better context since we tested both reciprocal hybrids and compared their performance to both parental populations on all three knotweed species. We found that the survival of the reciprocal hybrids is somewhat intermediate between those of the parental populations on all three knotweed species (Figure 2), which is a common outcome of intraspecific hybridization (Dingle et al., 1982; He et al., 2021; Szűcs et al., 2012; Tauber et al., 1986). This means that overall, hybridization improved performance compared to one parent and decreased performance compared to another parent. However, intermediate developmental success is better than no development at all (see Hokkaido psyllids on Japanese and Bohemian knotweeds).

Considering developmental success alone we concur with Grevstad et al. (2013) that the Kyushu population is best suited for releases on Bohemian and Japanese knotweeds and the Hokkaido population should only be used for releases on giant knotweed. However, the Kyushu population could also be used on giant knotweeds if the Hokkaido population is not available since they have relatively high‐developmental success on this species. In cases where cold adaptation traits from the Hokkaido population may be desirable, such as in Michigan, the release of hybrids might increase chances of overwintering success and should be considered as a viable alternative to either parental population.

An additional climate factor is photoperiod, which combined with temperature is used by most insects, including *A. itadori* to decide when to enter diapause (Danilevskii, 1965; Grevstad et al., 2022). Native species are locally adapted to use the cues from shortening daylength to prepare for winter and they will switch from a reproductive phase to a non‐reproductive phase at a critical photoperiod (Danilevskii, 1965; Masaki, 1999). The Hokkaido population that was derived from collections made at 42.6° latitude enters its non‐reproductive stage at a longer critical photoperiod than the Kyushu psyllids originating from a latitude of 32.8° N. This means that Hokkaido psyllids will diapause earlier than their Kyushu counterparts in the field (Grevstad et al., 2022). This appears to be a desirable trait in southern Michigan which is located at the same latitude as Hokkaido. However, the Hokkaido population will have low survival on the prevailing Japanese and Bohemian knotweeds in southern Michigan. On the other hand, because of their shorter critical photoperiod, Kyushu psyllids may start a new generation later in the season that they cannot complete before cold temperatures set in.

In sum, based on mismatches in temperature, photoperiod, and host plant availability neither the Kyushu nor the Hokkaido population may be ideal for releases in southern Michigan. Given that the hybrids between these two populations are able to develop and lay eggs on any of the three knotweed species and that they will have a mix of genotypes from both parental populations it is likely that there will be individuals with traits that can confer better survival and performance than those of either parent. Hybrids could also adapt faster to altered climates and photoperiod regimes because of their likely higher genetic diversity. We know from other weed biocontrol systems that critical daylength can evolve rapidly and that hybridization can alter the timing of diapause (Bean et al., 2012; Szűcs et al., 2012). For example, rapid evolution of the critical photoperiod was found in *Diorhabda carinulata* allowed the southward expansion of this agent used to control *Tamarix* spp. In the USA (Bean et al., 2012). In ragwort flea beetles (*Longitarsus jacobaeae*) used against the invasive *Jacobaea vulgaris* intraspecific hybridization altered the summer diapause response (Szűcs et al., 2012) and increased the biocontrol potential of hybrids in the field (Szűcs et al., 2019).

In addition, as a hybrid species, Bohemian knotweeds can possess a greater degree of genetic diversity, which has been proposed as a main characteristic of this species' invasive potential (Clements et al., 2016; Parepa et al., 2014). In such a case where genotypes of a target species are diverse, hybrids may be well suited in that they maintain a greater degree of diversity of their own compared to the parental populations, potentially benefitting establishment probability through the increased likelihood of adaptive evolution (Szűcs et al., 2017).

The release program of *A. itadori* in North America is still in its early stages, so monitoring of initial Kyushu and Hokkaido field populations will be essential to assess the establishment success and control potential of these two parental populations. In addition, a new population of *A. itadori* was collected in Niigata prefecture in Japan on the island of Honshu in 2019, called the Murakami population (Camargo et al., 2022). This population performs best on Bohemian knotweed and based on collection location its climate adaptation may be somewhat intermediate between the Kyushu and Hokkaido populations (Camargo et al., 2022). The first releases of the Murakami population were conducted in the Netherlands in 2020 (Camargo et al., 2022). Releases in North America may commence soon providing an opportunity to compare establishment rates of the three different psyllid populations and the hybrids discussed above to further evaluate the merits of hybridization in this system.

## AUTHOR CONTRIBUTIONS

**Andrew Yoshimoto:** Conceptualization (equal); formal analysis (equal); investigation (lead); methodology (equal); visualization (lead); writing – original draft (supporting); writing – review and editing (supporting). **Marianna Szűcs:** Conceptualization (equal); formal analysis (equal); funding acquisition (equal); methodology (equal); project administration (lead); resources (lead); supervision (lead); visualization (supporting); writing – original draft (lead); writing – review and editing (lead).

## FUNDING INFORMATION

Michigan Department of Natural Resources Michigan Invasive Species Grant Proposal (award # IS19‐5003). M.S. was supported by the United States Department of Agriculture National Institute of Food and Agriculture (USDA NIFA) Hatch projects 1017601 and 1018568.

## CONFLICT OF INTEREST STATEMENT

The authors declare no conflict of interest.

## Data Availability

Data for this study is available at https://doi.org/10.5061/dryad.bvq83bkgs
